# Design, Synthesis and Preclinical Assessment of ^99m^Tc-iFAP for In Vivo Fibroblast Activation Protein (FAP) Imaging

**DOI:** 10.3390/molecules27010264

**Published:** 2022-01-01

**Authors:** Diana Trujillo-Benítez, Myrna Luna-Gutiérrez, Guillermina Ferro-Flores, Blanca Ocampo-García, Clara Santos-Cuevas, Gerardo Bravo-Villegas, Enrique Morales-Ávila, Pedro Cruz-Nova, Lorenza Díaz-Nieto, Janice García-Quiroz, Erika Azorín-Vega, Antonio Rosato, Laura Meléndez-Alafort

**Affiliations:** 1 Department of Radioactive Materials, Instituto Nacional de Investigaciones Nucleares, Ocoyoacac 52750, Mexico; sarahitrujillo097@hotmail.com (D.T.-B.); blanca.ocampo@inin.gob.mx (B.O.-G.); clara.cuevas@inin.gob.mx (C.S.-C.); pedro.cruz.comecyt@inin.gob.mx (P.C.-N.); erica.azorin@inin.gob.mx (E.A.-V.); 2 Faculty of Chemistry, Universidad Autónoma del Estado de México, Toluca 50180, Mexico; gevillegas@live.com.mx (G.B.-V.); emoralesav@uaemex.mx (E.M.-Á.); 3 Department of Reproductive Biology, Instituto Nacional de Ciencias Médicas y Nutrición Salvador Zubirán, Ciudad de México 14080, Mexico; lorenzadiaz@gmail.com (L.D.-N.); janice.garciaq@incmnsz.mx (J.G.-Q.); 4 Department of Surgery, Oncology and Gastroenterology, University of Padua, Via Gattamelata 64, 35138 Padua, Italy; antonio.rosato@unipd.it; 5 Department of Immunology and Molecular Oncology Diagnostics, Veneto Institute of Oncology IOV-IRCCS, Via Gattamelata 64, 35138 Padua, Italy; laura.melendez.alafort@iov.veneto.it

**Keywords:** fibroblast activation protein, FAP inhibitors, technetium-99m, HYNIC-iFAP

## Abstract

Fibroblast activation protein (FAP) is expressed in the microenvironment of most human epithelial tumors. ^68^Ga-labeled FAP inhibitors based on the cyanopyrrolidine structure (FAPI) are currently used for the detection of the tumor microenvironment by PET imaging. This research aimed to design, synthesize and preclinically evaluate a new FAP inhibitor radiopharmaceutical based on the ^99m^Tc-((R)-1-((6-hydrazinylnicotinoyl)-D-alanyl) pyrrolidin-2-yl) boronic acid (^99m^Tc-iFAP) structure for SPECT imaging. Molecular docking for affinity calculations was performed using the AutoDock software. The chemical synthesis was based on a series of coupling reactions of 6-hidrazinylnicotinic acid (HYNIC) and D-alanine to a boronic acid derivative. The iFAP was prepared as a lyophilized formulation based on EDDA/SnCl_2_ for labeling with ^99m^Tc. The radiochemical purity (R.P.) was verified via ITLC-SG and reversed-phase radio-HPLC. The stability in human serum was evaluated by size-exclusion HPLC. In vitro cell uptake was assessed using N30 stromal endometrial cells (FAP positive) and human fibroblasts (FAP negative). Biodistribution and tumor uptake were determined in Hep-G2 tumor-bearing nude mice, from which images were acquired using a micro-SPECT/CT. The iFAP ligand (*Ki* = 0.536 nm, AutoDock affinity), characterized by UV-Vis, FT-IR, ^1^H–NMR and UPLC-mass spectroscopies, was synthesized with a chemical purity of 92%. The ^99m^Tc-iFAP was obtained with a R.P. >98%. In vitro and in vivo studies indicated high radiotracer stability in human serum (>95% at 24 h), specific recognition for FAP, high tumor uptake (7.05 ± 1.13% ID/g at 30 min) and fast kidney elimination. The results found in this research justify additional dosimetric and clinical studies to establish the sensitivity and specificity of the ^99m^Tc-iFAP.

## 1. Introduction

Fibroblast activation protein (FAP) is a type II serine protease that cleaves peptides after proline residues with endopeptidase and dipeptidyl-peptidase activities. FAP is primarily expressed on activated stromal fibroblasts present in most human epithelial tumors but not in normal fibroblasts [1].

Specific FAP inhibitors were initially developed as potential anticancer drugs [2,3,4]. The most relevant are two groups of highly selective compounds, one based on a quinoline-cyanopyrrolidine structure and the other based on pyrrolidine-boronic acid also known as proline-boronic acid (boroPro). Regarding the first group, Jansen et al. reported the synthesis of 39 new FAP inhibitors to assess the 4-quinolinoyl-Gly-cyanopyrrolidine activity [5]; they found that FAP forms covalent bonds with the nitrile group [6]. The authors also reported that derivatives containing N-pyridines produce FAP inhibitors recognized by other cleavage enzymes, such as prolyl-oligopeptidase (PREP) and dipeptidyl-peptidases (DPP), while the molecules containing the quinolinoyl fragment showed a higher affinity for FAP. Furthermore, they found that the addition of fluorine to the cyanopyrrolidine ring fragment improved the molecular recognition by FAP. When the glycine residue was substituted with other amino acids, the potency of the FAP decreased significantly and the affinity for PREP increased; thus, the authors identified the N-(4-quinolinoyl)-glycyl-(2-cyanopyrrolidine) scaffold as the best FAP inhibitor.

Additionally, Poplawski et al. synthesized and characterized more than 20 PREP and FAP boroPro inhibitors [7]. The authors reported an increased affinity for FAP when the pyridine nitrogen was protonated to form a hydrogen bond with the Glu-204 amino acid of FAP, not present in PREP. Based on these results, Poplawski et al. established that N-(pyridine-4-carbonyl)-D-Ala-boroPro was an efficient fibroblast activation protein inhibitor.

Despite the large number of synthesized and characterized FAP inhibitors, only a limited number of them have been radiolabeled. In 2015, the first radiolabeled FAP inhibitor, based on boronic acid (^125^I-MIP-1232) was reported [8].

In 2018, Lindner and Loktev reported the conjugation of DOTA (2,2′,2″,2‴-(1,4,7,10-tetraazacyclododecane-1,4,7,10-tetrayl)tetraacetic acid) to 4-quinolinoyl-Gly-cyanopyrrolidine, with the aim to obtaining radioligands for either diagnostic or therapeutic applications [9,10]. The authors developed fifteen FAP inhibitor derivatives (FAPI). The different FAPI molecules were radiolabeled with ^177^Lu, ^90^Y or ^68^Ga. From them, ^68^Ga-FAPI-02 and ^68^Ga-FAPI-04 proved to be the most suitable agents for diagnostic applications. Subsequently, the same authors reported other derivatives of the same framework numbered consecutively from FAPI-21 to FAPI-55 [11]. The first FAP inhibitors used in humans for PET scans were ^68^Ga-FAPI-02 and ^68^Ga-FAPI-04 [12,13]. The substitution of DOTA for 1,4,7-triazacyclononane-N,N′N′-triacetic acid (NOTA) in FAPI-12 produced the derivative FAPI-74, which can be labeled with ^68^Ga or ^18^F [14]. FAPI-35 was developed for labeling with ^99m^Tc and possibly ^188^Re through carbonyl technology [15].

The diagnostic radiopharmaceuticals that inhibit FAP, so far developed and applied in clinical studies, use ^68^Ga or ^18^F attached to derivatives of 4-quinolinoyl-Gly-cyanopyrrolidine, which are radiopharmaceuticals for PET, and only one study has used a derivative of ^99m^Tc, also bound to quinoline-cyanopyrrolidine. However, SPECT studies still represent more than 70% of the total in nuclear medicine due to their lower cost and greater availability of equipment. For SPECT imaging, the most widely-used radionuclide is ^99m^Tc and there is no publication devoted to the design and study of radiopharmaceuticals for FAP imaging based on boroPro derivatives.

Considering that the 6-hidrazinylnicotinic acid (HYNIC) contains two nitrogen atoms of the hydrazine group that could function as additional anchoring sites in the active site of FAP, as well as HYNIC’s ability to function as a ^99m^Tc chelator, the objective of this research was to design, synthesize and preclinically evaluate, a new FAP inhibitor radioligand based on the ^99m^Tc-HYNIC-D-alanine-boroPro (^99m^Tc-iFAP) structure.

## 2. Results and Discussion

### 2.1. Molecular Docking

**Figure 1:**
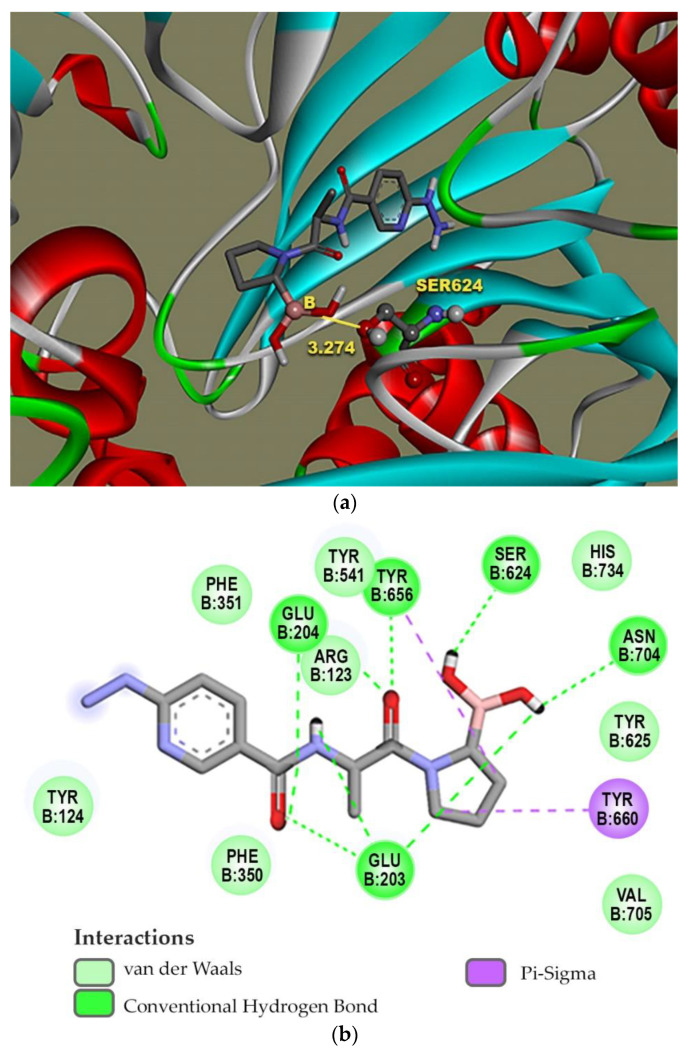
Molecular modeling: (**a**) a FAP ligand-binding cavity for iFAP and (**b**) map of the interactions between iFAP and FAP in the ligand binding pocket.

The design of the iFAP molecule [((R)-1-((6-hydrazinylnicotinoyl)-D-alanyl) pyrrolidin-2-yl) boronic acid] was carried out by molecular docking, which was compared in affinity and inhibition constant (Ki) with two of the most representative structures of the boroPro family reported in the literature: N-acyl-Gly-boroPro [4] and N-(pyridine-4-carbonyl)-D-Ala-boroPro [7] (Table 1). In agreement with the interaction map of iFAP and the amino acid residues of the active center of FAP obtained in the molecular coupling study (Figure 1), the increase in affinity of the iFAP, with regard to the N-(pyridine-4-carbonyl)-D-Ala-boroPro, can be attributed to the presence of hydrazine nitrogens in the HYNIC. These favor van der Waals-type interactions and hydrogen bonds of the iFAP molecule in its coupling to the active center of FAP, mainly via the residues Glu-203, Glu-204, Phe-350 and Phe-351, as well as a greater proximity for the interaction with Ser-624, which functions as the enzyme’s active center [4].

### 2.2. Chemical Synthesis

As described in detail in the methodology section, the chemical synthesis was based on a series of coupling reactions of 6-hidrazinylnicotinic acid (HYNIC) and D-alanine to a boronic acid derivative [16]. The overall yield was 62% and the chemical purity was 92%, as determined by both HPLC and UPLC.

### 2.3. Chemical Characterization

#### 2.3.1. iFAP

**Figure 2:**
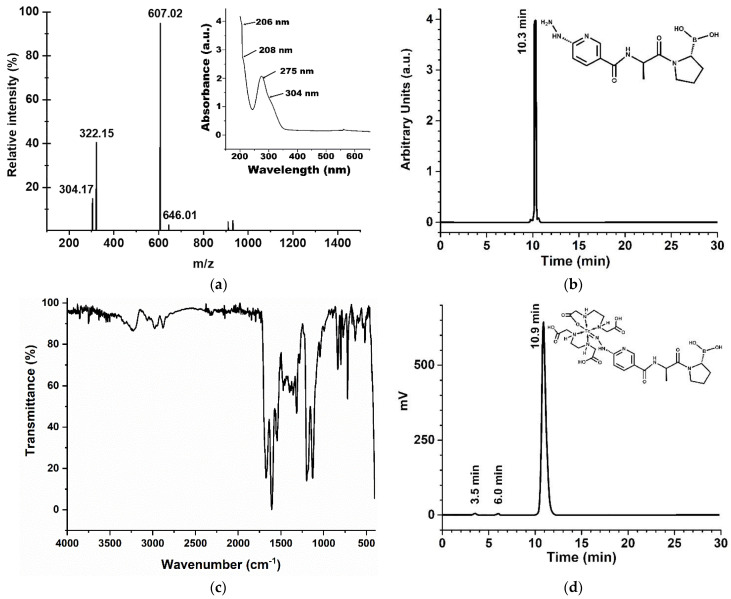
Chemical characterization of the iFAP ligand: (**a**) mass spectrum (iFAP in DMSO) and UV-Vis spectrum (inset) of iFAP in aqueous solution, pH 6.5; (**b**) reversed-phase UV-HPLC, 0.05 mg/mL of iFAP in aqueous solution, injection of 20 µL; (**c**) infrared spectrum (ATR-FTIR) of iFAP, lyophilized powder; and (**d**) reversed-phase radio-HPLC chromatogram of ^99m^Tc-iFAP (inset: proposed structure) in 0.9% NaCl solution, 37 MBq/mL, injection of 20 µL.

The UV-vis spectrum of the iFAP reveals shoulders at 206 nm and 208 nm, which are assigned to the n→σ* transitions from hydrazine (C-NH-NH_2_). The strong band at 275 nm and the shoulder at 304 nm are associated with π→π* and n→π* transitions (–C=C– and –C=N– groups) of the pyridine ring (Figure 2a, inset). Reversed-phase HPLC analysis of the lyophilized white solid showed a chemical purity of 92% (t_R_=10.3 min) (Figure 2b). The final product presented the expected mass spectrum: *m*/*z* 322 (calcd. 321) [M + H]^+^, *m*/*z* 646 (calcd. 321) 2× [M + 2H]^+^. Other signals observed at 304 and 607 *m*/*z* correspond to the formation of boronate esther ions assigned to [M + H–H_2_O]^+^ and 2 × [M + H–H_2_O]^+^, respectively (Figure 2a). The ^1^H–NMR spectroscopy corroborated the presence of major structures in the molecule, for instance, the pyridine signals could be detected from 8.5 to 6.7 ppm establishing the presence of a HYNIC ligand, while signals from 3.1 to 3.3 supported the proline-boronic acid detection, ^1^H–NMR (300 MHz, DMSO), δ (ppm): 8.5–6.7 (s, 1H, –CH_6_–, arom. pyridine), 9.7 (s, 1H, –NH–NH–), 8.3 (s, 1H, –CH_2_–NHCO), 3.1–3.3 (m, 1H, CH_2_CHB).

In the FT-IR spectrum of the iFAP shown in Figure 2c, the bands at 1359 cm^−1^ and 1316 cm^−1^ associated with the B-O vibration of boronic acid [17], and the band at 3335 cm^−1^ associated with the vibrations (-N-H)υ of the pyridine ring, could be observed. The band at 3240 cm^−1^ was attributed to the vibrations generated by both the O-H groups of boric acid and the N-H vibration of the hydrazine amine. The band at 2950 cm^−1^ corresponds to the υ_s_ vibration, while the band at 2880 cm^−1^ indicates the υ_as_-vibration of -C-H(CH_3_) of the alanine amino acid. The bands found in the region of 2978 cm^−1^ and 2835 cm^−1^ correspond to the (-CH_2_)υ_s_ and (-CH_2_)υ_as_ vibrations of the methylenes in the proline ring [18], respectively. The band at 1675 cm^−1^ was associated with the C=O vibration of the tertiary amide, while the band at 1608 cm^−1^ was attributed to the C=O vibrations of the secondary amide. The bands between 1450-1600 cm^−1^ were assigned to the aromatic C-C=C ring stretch; those of 3064 cm^−1^ and 3032 cm^−1^ were attributed to the aromatic C-H stretch vibrations, and the bands at 1128 cm^−1^ and 1199 cm^−1^ were associated with aromatic C-H in-plane bending, while the bands between 900–670 cm^−1^ indicate aromatic C-H out-of-plane bending; all of them are a result of the presence of the pyridine ring [19]. Bands between 1450–1600 cm^−1^ are also correlated with C-N and C-N-H amide II stretching vibrations.

#### 2.3.2. ^99m^Tc-iFAP

After reconstitution of the iFAP lyophilized formulation with a ^99m^TcO_4_Na solution, the ^99m^Tc-EDDA/HYNIC-iFAP (^99m^Tc-iFAP) radiopharmaceutical was obtained with a radiochemical purity greater than 98%, as determined by reversed-phase HPLC (Figure 2d). As can be observed in Figure 2d the retention times (t_R_) for ^99m^TcO_4_Na, ^99m^Tc-EDDA/tricine, and ^99m^Tc-iFAP were 3.5, 6.0 and 10.9 min, respectively. Additionally, radiochemical purity determined by instant thin layer chromatography was 99%. It should also be noted that the radiopharmaceutical remains stable with a radiochemical purity greater than 97% after 24 h of labeling.

It is important to mention that the chromatograms are usually obtained with the UV-Vis detector and the radiometric detector. After injection, the radiotracer is first identified by the UV-Vis device and after 0.6 min (0.6 mL, 1 mL/min) it is sensed by the radioactive detector. Correspondence of the retention times between the iFAP peak observed in the UV-vis chromatogram (Figure 2b) and the ^99m^Tc-iFAP peak in the radio-chromatogram (Figure 2d) is usually accepted as a proof of the chemical identity of the radiotracer [20].

Although crystallographic characterization of ^99m^Tc-HYNIC-based radioligands has not yet been reported, studies on Tc-6-hydrazinylnicotinoyl complexes have confirmed the configuration of a stable [Tc = NNR] core as the molecular identity [21,22]. It is also well-known that HYNIC satisfies only part of the technetium coordination requirements and that with two EDDA molecules as coligands, high in vitro and in vivo stable complexes are obtained [23,24,25,26].

### 2.4. In Vitro Evaluation of ^99m^Tc-iFAP

#### 2.4.1. Serum Stability and Protein Binding

The ^99m^Tc-iFAP incubated during 30 min, 3 h and 24 h with fresh human serum at 37 °C, remained stable, as established by size-exclusion radio-HPLC. Chromatographic profiles were obtained using two different detector systems, the UV-vis detector, and a radiometric detector. The retention time (UV-vis chromatogram) of proteins and cysteine was from 4 to 7 min and 12.3 min, respectively, while ^99m^Tc-iFAP and ^99m^TcO_4_Na retention times were 8.7 min and 11.5 min (radiochromatogram), respectively. During the sample analyses at different times, a shift in the radioactivity at a higher molecular weight associated with the retention time of the proteins (4–7 min) was considered as the percentage of the binding of the radiopharmaceutical to the serum proteins. Therefore, in vitro stability tests of ^99m^Tc-iFAP in human serum demonstrated a serum protein binding of 0.3 ± 0.1%, 0.9 ± 0.2% and 2.1 ± 0.3% at 30 min, 3 h and 24 h, respectively, as well as high radiochemical stability (>95% at 24 h). No radioactivity associated with cysteine or peaks of lower molecular weight was obtained. These findings indicate that the transchelation of ^99m^Tc to cysteine does not occur, as well as indicating an absence of other radiolabeled catabolites because of the enzymatic degradation of ^99m^Tc-iFAP in the serum. It is important to mention that for the evaluation of radiochemical stability, the percentage of recovered activity was also considered and calculated, which made it also possible to determine the amount of ^99m^Tc-colloid as activity retained in the column (0.2%).

#### 2.4.2. Tumor Stroma Binding

**Figure 3:**
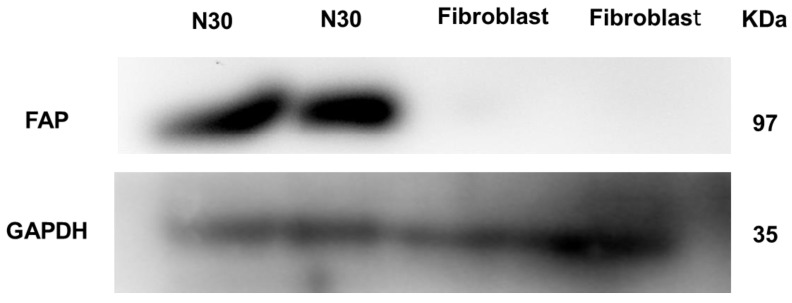
Western blot assay showing expression of FAP in N30 (primary human stromal endometrial cells) and negative FAP expression in human fibroblasts. GAPDH was used as a control.

**Figure 4:**
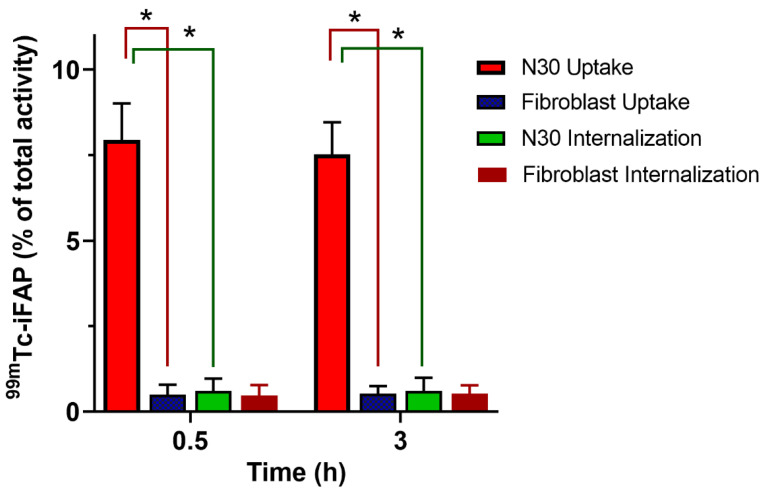
In vitro ^99m^Tc-iFAP uptake and internalization in N30 (primary human stromal endometrial cells, FAP positive) and by human fibroblasts (FAP negative control) (*n* = 6). * Significant difference, *p* < 0.05; *t*-student.

Since FAP is a highly-expressed membrane-anchored peptidase by cancer-associated fibroblasts in the tumor stroma of almost all types of malignant epithelial tumors, it is not possible to assess the affinity of ^99m^Tc-iFAP using non-transfected cancer cells. The use of a primary cancer cell culture, obtained through a biopsy procedure, is an option, as there is tumor stroma present. The N30 cells used in this research are primary human stromal endometrial cells [27]. The FAP expression in N30 stromal cells was confirmed by a Western blot assay employing an anti-FAP monoclonal antibody and human fibroblasts as a negative control (Figure 3). Accordingly, in cell uptake and internalization assays, we found that N30 showed an increased ^99m^Tc-iFAP cellular uptake when compared to fibroblasts (Figure 4, *p* < 0.05). Of note, the ^99m^Tc-iFAP internalization rate was rather low in the N30 cells (Figure 4), confirming that radiopharmaceutical uptake is associated with the expression of FAP in the membrane of N30 stroma cells.

**Figure 5:**
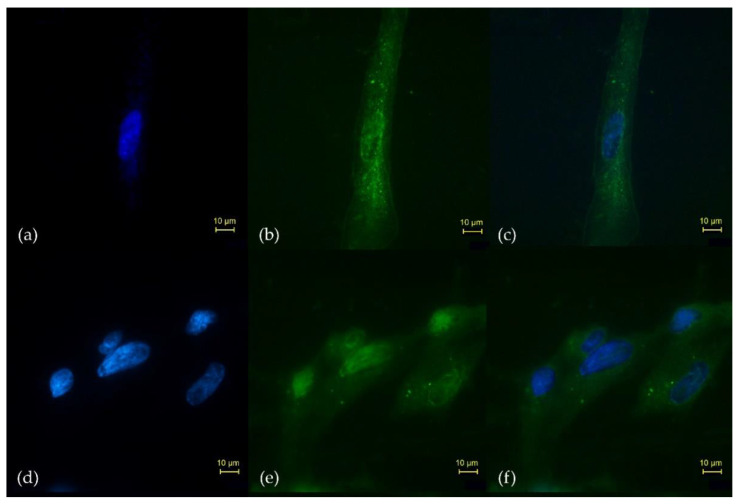
Microscopic fields (40×) of the in vitro uptake and internalization in the N30 cells (FAP-positive) of (**b**) Lu_2_O_3_-iFAP and (**e**) Lu_2_O_3_ nanoparticles, detailing (**a**,**d**) the Hoechst-stained nucleus, and the merged images of nuclear and N30 cells treated with (**c**) Lu_2_O_3_-iFAP or (**f**) Lu_2_O_3_ (control). Observe the qualitative difference regarding the number of lutetium oxide nanoparticles (bright spots) between the Lu_2_O_3_-iFAP (**b**) and nanoparticles without iFAP (**e**) in the membrane and cytoplasm of N30 cells.

The iFAP ligand was covalently attached on the surface of lutetium oxide nanoparticles (Lu_2_O_3_-iFAP colloidal solution), which had a significant uptake and were significantly internalized in the N30 cells (Figure 5b); most likely due to a specific molecular recognition (iFAP/FAP) as the first interaction, and afterwards, as a passive internalization mechanism, due to the inherent nature of nanoparticles (50–100 nm). When the Lu_2_O_3_ nanoparticles were not conjugated to the iFAP ligand, cellular uptake and internalization was scarce (Figure 5e), which represents qualitative evidence of specific iFAP recognition (Figure 5).

### 2.5. In Vivo Evaluation of ^99m^Tc-iFAP

**Figure 6:**
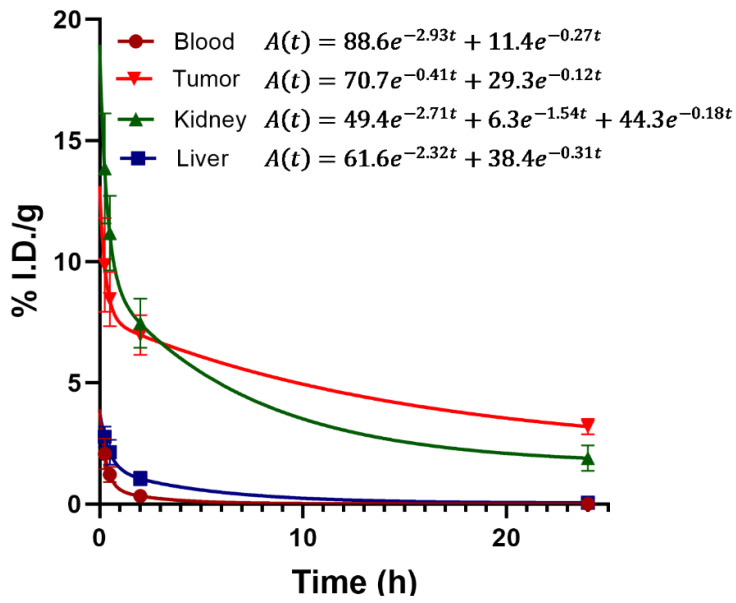
Biokinetic models of ^99m^Tc-iFAP in the four main target tissues: blood, tumor, kidney and liver. As can be observed, blood showed the fastest elimination and tumor, the lowest.

The biodistribution assessment of ^99m^Tc-iFAP in nude mice with induced liver cancer tumors (Hep-G2 cells) showed a tumor uptake of 7.05% and 5.18% of the activity administered per gram of tissue (% ID/g) at 30 min and 2 h post-injection, respectively, with rapid elimination mainly via the kidneys (Table 2). Biokinetic analysis in the four main target tissues/organs of the radiopharmaceutical, blood, liver, kidneys, and tumor, are shown in Figure 6. The results indicated a very rapid blood clearance, where 88.6% of the radiotracer was eliminated with a half-life (fast) of 0.24 h (t_1/2_ = ln2/2.93) and 11.4% with a half-life (slow) of 2.57 h (t_1/2_ = ln2/0.27). The biokinetic model of the kidneys showed a triexponential elimination behavior (three elimination phases), where 49.4% of the activity was eliminated with a half-life (fast) of 0.26 h (t_1/2_ = ln2/2.71), 6.3% with a half-life of 0.45 h (t_1/2_ = ln2/1.54), and 44.3% had a mean elimination time (slow) of 3.85 h (t_1/2_ = ln2/0.18). Of all the elimination constants shown in the biokinetic model of the main target organs, the tumor had the lowest fast elimination constant (λ_eff_ = 0.41), as well as the lowest slow elimination constant (λ_eff_ = 0.12), regarding all the other organs; therefore, ^99m^Tc-iFAP showed the longest residence times in tumors (Figure 6).

**Figure 7:**
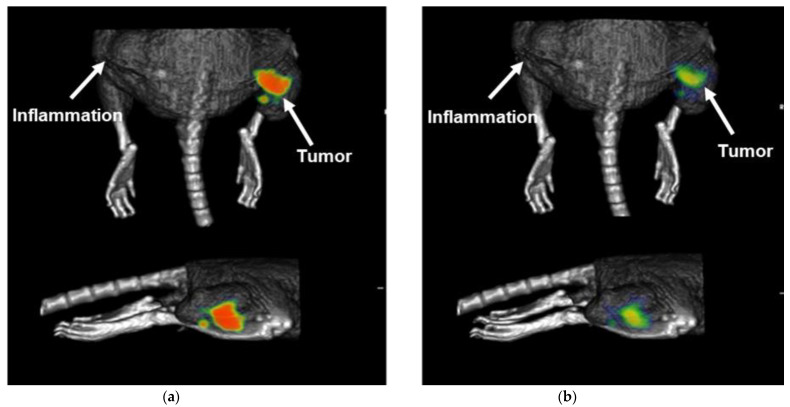
Micro-SPECT/CT imaging of ^99m^Tc-iFAP in a nude mouse with an induced Hep-G2 tumor at (**a**) 30 min (SUV max of 5.6, right thigh), and (**b**) 24 h (SUVmax of 2.4, right thigh) after radiopharmaceutical administration. It is worth noting the significant radiopharmaceutical uptake in the right thigh of the mouse where the cancer cells were inoculated (positive control), whereas, in the left thigh, where an inflammatory process was induced with killed bacteria (negative control), no radiotracer uptake was observed.

The cold ligand (iFAP) did not show adverse effects when administered at a dose of 40 mg/kg to healthy laboratory Balb-C mice. Micro-SPECT/CT imaging of the ^99m^Tc-iFAP in nude mice with induced Hep-G2 lesions showed high tumor uptake (SUVmax = 5.0 ± 1.2) at 30 min after administration (Figure 7a), with a significant tumor retention at 24 h (SUVmax = 2.7 ± 0.6) (Figure 7b), as observed in the right thighs of mice, where the cancer cells were inoculated (positive control), while the left thighs, where an inflammatory process was induced with killed bacteria (negative control), no radiotracer uptake was observed (Figure 7).

Recently, several PET radiotracers have been reported for the targeting of the FAP protein in the tumor stroma; particularly, on the surface of cancer-associated fibroblasts [4]. In this research, a FAP inhibitor, based on a boroPro molecule, labeled with ^99m^Tc (^99m^Tc-iFAP) for in vivo detection of the microenvironment of tumors by SPECT in nuclear medicine, is reported for the first time. Although a previous report established that the presence of a nitrogen atom in the 4-position of pyridine [N-(Pyridine-4-carbonyl)-DAla-boroPro] was essential for FAP selectivity [7], the incorporation of HYNIC instead of pyridine-4-carbonyl demonstrated a selectivity for malignant tumors. It is possible that the hydrazine group of HYNIC affects the molecule orientation and provides additional van der Waals-type interactions and hydrogen bonds of iFAP in its coupling to the active center of FAP.

The biokinetic profile of the ^99m^Tc-iFAP is comparable with those reported for ^18^F-FAPI-46, ^68^Ga-FAPI-02 and ^99m^Tc-FAPI-34 with regards to the fast whole-body elimination [9,10,12,13]. In the same sense, the retention of ^99m^Tc-iFAP in the tumor microenvironment was lower than that observed for ^99m^Tc-iPSMA or ^99m^Tc-Tyr^3^-octreotide, usually employed in the targeting of receptors overexpressed in cancer cells and suitable for labeling with ^177^Lu or ^225^Ac for theranostic applications [23,25,26]. Due to the relatively fast tumor elimination, ^177^Lu and ^225^Ac could not be appropriate radionuclide pairs for FAPI derivatives. Even so, FAPI-46 labeled with ^90^Y, ^153^Sm, or ^177^Lu has been reported for therapeutic applications [28,29,30]. To overcome this limitation, new FAPI-dimers are under evaluation [31]. As Lindner et al. have proposed [13], it is also possible to use the ^99m^Tc/^188^Re pair for theranostics targeting FAP, due to the high dose rate produced by ^188^Re as a consequence of its shorter half-life (16.9 h) compared to ^177^Lu (162 h). In this context, ^99m^Tc-FAPI-34 has an advantage over ^99m^Tc-iFAP, because the former is based on the tricarbonyl labeling technology, which is proven for use with ^188^Re, while with HYNIC, the labeling with ^188^Re is not feasible. Nevertheless, the iFAP ligand has the ability to detect with a high sensitivity, the expression of the FAP in the tumor microenvironment. Therefore, the coupling of several iFAP molecules on the surface of lutetium oxide nanoparticles activated by neutron irradiation (^177^Lu_2_O_3_-iFAP nanoparticles) would make it possible to have a stable colloidal solution for potential therapeutic applications, as we previously reported for ^177^Lu_2_O_3_-iPSMA [32].

The ^99m^Tc-iFAP preclinical results reported in this research have allowed the approval of preliminary clinical studies which are in progress and which have demonstrated a high sensitivity of the radiopharmaceutical to detect the tumor microenvironment of different types of cancer such as breast, lung, colorectal, gliomas, and cervical cancer using SPECT/CT imaging.

## 3. Materials and Methods

### 3.1. Materials

N-Boc-pyrrolidine, sec-butyllithium solution (1.4 M in cyclohexane), trimethyl borate, (1S,2S,3R,5S)-(+)-Pinanediol, Boc-Ala-OH, phenylboronic acid and Ethylenediamine-N,N′-diacetic acid (EDDA) were provided by Sigma-Aldrich Chemical Co. (St. Louis, MO, USA); succinimidyl-N-Boc-HYNIC was supplied by Synchem UG & Co (Felsberg-Altenburg, Germany); ^99m^Tc-pertechnetate was obtained from a ^99^Mo/^99m^Tc generator (ININ, Mexico); DMEM-F12 culture media was provided by ATCC (Manassas, VA, USA) and the anti-FAP monoclonal antibody by Invitrogen (Waltham, MA, USA); all of the other reagents were of analytical grade and were provided by Merck Millipore Co. (Burlington, MA, USA) and Gibco (Grand Island, NY, USA), as mentioned in each case; the N30cell line was obtained in the Wake Forest School of Medicine, originally donated by Dr. Taylor (Department of Obstetrics and Gynecology, Winston Salem, NC, USA); the Hep-G2 was supplied from ATCC (Atlanta, GA, USA). The human dermal fibroblasts were donated by Dr. Martínez-Gutiérrez from the “Facultad de Ciencias Químicas, Universidad Autónoma de San Luis Potosí, Mexico”.

### 3.2. Methods

#### 3.2.1. Molecular Docking

The structures for HYNIC-iFAP and other FAP inhibitors were generated in ChemDraw (.cdx format) and the 3D structure was exported in .pdb format through the Chem3D software. Using the AVOGADRO 1.2.0 molecular editor, the molecular geometry was optimized using a universal force field (UFF) due to the presence of a boron atom, with a total of 10,000 steps. Subsequently, a second optimization of the geometry was carried out using the semi-empirical software of Quantum Chemistry MOPAC2016 with a PM7 theory level, exporting the resulting spatial configuration to .pdb format.

The crystal structure of the alpha subunit of fibroblast activating protein (FAP) was obtained through the RSCB Protein Data Bank (PDB ID: 1Z68). In order to be able to use it as a receptor macromolecule in molecular coupling calculations, the molecule was edited in BIOVIA Discovery Studio 2021 to eliminate water molecules and residues manifested in X-ray diffraction, leaving only the main chain of amino acids in the .pdb file. Chain A was also eliminated from the dimer represented in the model, leaving only chain B. Since the three-dimensional model of the macromolecule involves a resolution of 2.60 Å, a homology modeling step was performed through the SWISS online platform, MODEL. The resulting structure was saved in .pdb format for use as a receiver.

Both the receptor and FAP inhibitor structures were prepared with the OPEN BABEL GUI 2006 library indicating the addition of missing hydrogens and their molecular optimization for a physiological pH (pH = 7.4), again generating the structures in .pdb format. The AutoDock Tools 1.5.6 software package was used to configure the receptor as a macromolecule and each of the ligands in separate files, exporting the files with a .pdbqt extension. The search box was configured in the hydrophobic site S1 around Ser-624 of the receiver. As the center, the XYZ coordinates 18,948, 10,676, 28,989, respectively, were established and a size of 90 was established in each axis of the box. Regarding the ligands, it was necessary to modify the file << AD4_parameters.dat >> to add the parameters for the boron atom available at http://mgldev.scripps.edu/pipermail/autodock/2009-March/005439.html (accessed on 16 May 2020).

atom_par B 4.08 0.180 12.052 -0.00110 0.0 0.0 0 -1 -1 0 # Non H-bonding

The execution of the protein-ligand molecular coupling was carried out with the AutoDock 4.2.6 package, previously calculating the necessary .map grids with the AutoGrid 4.2.6 tool. The log file with the results of the molecular coupling was viewed in AutoDock Tools, exporting the complex with the best affinity score to .pdb format. The visualization and analysis of distances and interactions were performed using the BIOVA Discovery Studio Visualizer 2021.

The calculation of the inhibition constant (*Ki*) was carried out via the following Equation (1):

(1) $$Ki=e^{\frac{affinity}{RT}}$$

where:

*Ki*: is the inhibition constant,

*affinity*: is the value given by AutoDock 4.2.6,

*R*: is the universal gas constant with a value of 0.00198179 kcal/mol K,

*T*: is the temperature in Kelvin scale used in AutoDock 4.2.6 (298.15 K).

#### 3.2.2. Synthesis of iFAP

**Figure 8:**
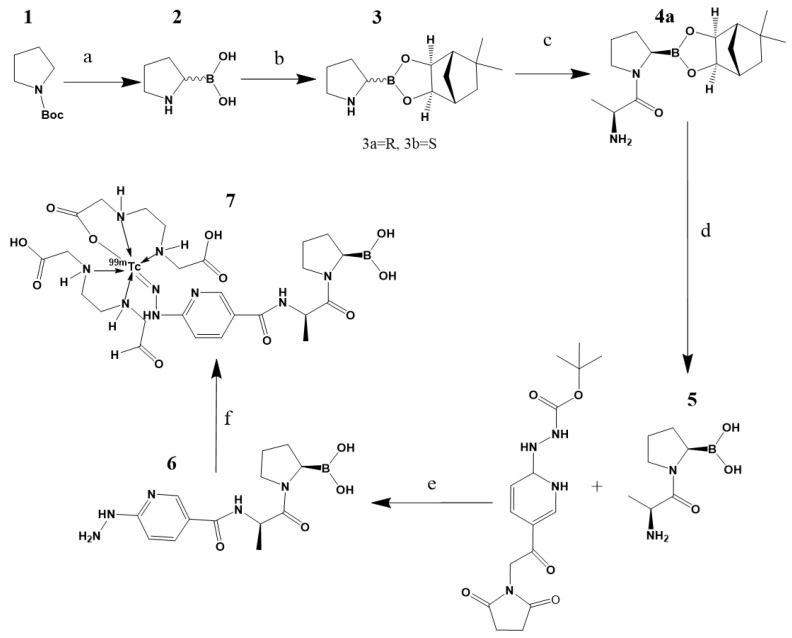
Reagents: (**a**)(1) s-buLi, Et_2_O, TMDA, −40 °C, 3 h, (2) B(OCH_3_)_3_, (3) NaOH, (4) HCl; (**b**)(1) (+)pinanediol, Et_2_O, 2 h; (**c**) (1) Boc-D-alanine, CH_2_CL_2_, HOBT, EDC, 30 min, 0 °C, (2) **3a,** NMM, 8 h, (3) TFA, (4)H_2_O, Na_2_CO_3_, KHSO_4_; (**d**) (1) **4a,** H_2_O, (2) PhB(OH)_2_, hexane, 24 h; (**e**) (1) Succinimidyl-N-Boc-hynic, DIPEA, HATU, DMF, 15 min, (2) **5**, 2 h, (3) TFA; and (**f**) (1) EDDA/tricine (2) **6**, (3) ^99m^TcO_4_Na/SnCl_2_, 15 min, 92 °C; and **7**, proposed structure of the final product.

For the synthesis of iFAP (Figure 8), Boc-pyrrolidine (10 mmol) was reacted with a solution of sec-butyllithium (12 mmol) dissolved in 20 mL of ethyl ether and 3 mL of tetramethylethylenediamine (20 mmol) at −40 °C during a 3 h period, followed by the addition of trimethyl borate (30 mmol). The reaction was stopped via a temperature increase (room temperature) and the addition of water (30 mL). Afterwards, three volumes (3 × 30 mL) of 2 M sodium hydroxide were added for methoxy group hydrolysis and formation of the boronate salt; the free boronic acid (compound 2) was obtained via the adition of 2 M hydrochloric acid until a pH = 3 was reached and extracted with ethyl acetate. Compound 2 was reacted with (+) pinanediol (20 mmol) in 60 mL of ethyl ether for 2 h. The purification of the product was completed via a silica gel column (hexane:ethyl acetate 6:1) to obtain the mixture diastereomers R and S. The Boc group was eliminated via acid hydrolysis using ethyl acetate/dry HCl to obtain compound 3. The R configuration was separated via the use of a silica HPLC column (10 μm particle size; L × I.D. 25 cm × 4.6 mm; mobile phase: ethyl ter-butyl ether:hexane 1:9) at 220 nm, where R was the second elution product.

The compound **3a** (10 mmol) was placed with N-methylmorpholine (20 mmol) and added to the mixture of Boc-D-alanine (10 mmol) in 100 mL of dichloromethane with hydroxybenzotriazole (10 mmol) and ethyl-3-(3-dimethylamonipropyl)carbodiimide (13 mmol) at 0 °C. The reaction was left stirring for 8 h at room temperature. Afterwards, the product was washed with water, 1 M sodium carbonate and 1 M dipotassium hydrogen phosphate, and the organic layer was purified via a silica gel column (ethyl acetate). Finally, the Boc group was eliminated with ether/dry HCl to obtain **4a.**

For the elimination of (+)pinanediol, **4a** (10 mmol) in 100 mL of 2 M hydrochloric acid was reacted with phenylboronic acid (15 mmol) in 100 mL of hexane. The two-phase suspension was left stirring for 24 h with a fresh supply of hexane every 4 h. Posteriorly, the aqueous phase was separeted by HPLC (water) to obtain **5**. At this point, the yield of the general procedure was in agreement with that reported by Coutts et al. [16].

In our procedure, succinimidyl-N-Boc-HYNIC (10 mmol) was mixed with N,N-diisopropylethylamine (100 mmol) and HATU (30 mmol) in 2 mL of dimethylformamide. The solution was stirring for 15 min at room temperature and **5** (10 mmol) was added and stirred for 2 h. Finally, Boc deprotection was performed using 2 M trifluoroacetic acid. The final product, (R)-HYNIC-D-Ala-pyrrolidine-boronic acid (**6**), was purified via a reversed-phase HPLC column (Discovery C-18, 5 μm particle size; L × I.D, 25 cm × 10 mm. Mobile phase: water:acetonitrile 40:60 *v*/*v*) for lyophilization. The overall yield was 62%.

#### 3.2.3. Chemical Characterization

The ultraviolet-visible spectrum (UV-vis) was obtained in an aqueous solution (iFAP, 0.2 mg/mL, pH 6.5) in the range of 200–800 nm with the use of a 1 cm quartz cuvette on a Lambda Bio spectrometer (PerkinElmer: Waltham, MA, USA).

The Fourier transform infrared (FT-IR) spectrum was obtained from the iFAP lyophilized powder. The spectrum was obtained in the range of 4000–400 cm^−1^ from 40 scans and a 1 cm^−1^ resolution on a PerkinElmer System 2000 spectrometer (Pike Technologies) with an ATR platform (Attenuated Total Reflection Fourier Transform Infrared). A ^1^H–NMR spectrum of iFAP in DMSO was obtained at 300 MHz, using tetramethylsilane as the internal reference.

The mass spectrum was obtained in the range of 100 to 1500 *m*/*z* via electrospray UPLC-mass spectroscopy (SQD-MS) using an Xbridge BEH C18 column (3.5 µm, 4.6 mm × 150 mm), ESI positive ionization mode, cone voltage of 5 V and Waters MassLynx 4.1. sotfware, (Waters, London, UK). For the analysis, 10 µL of iFAP (1mg/mL) were injected, mobile phase A (0.1% trifluoroacetic in water) and mobile phase B (0.1% trifluoroacetic acid in acetonitrile) were applied with an elution flow of 1 mL/min. The elution linear gradient was: A-100% for 3 min, changed to A-50% over 10 min and maintained for 10 min, changed A-30% over 3 min and returned to A-100% over 4 min.

#### 3.2.4. Preparation of ^99m^Tc-iFAP

A sterile freeze-dried kit formulation containing EDDA (10 mg), tricine (20 mg), SnCl_2_ (20 µg), mannose (50 mg) and iFAP (50 µg) was prepared under the same conditions previously reported for HYNIC-iPSMA [26].

For radiolabeling, the lyophilized formulation was reconstituted with 1 mL of a 0.2 M phosphate buffer and 1 mL of ^99m^TcO_4_Na (740–1110MBq). The reaction was incubated in a block heater for 15 min at 92 °C. The radiochemical purity was 98% as determined by radio-HPLC (Discovery C18; 5 μm particle size; L × I.D. 25 cm × 4.6 mm; linear gradient: 0.1% TFA/water (A) and 0.1% TFA/acetonitrile (B) flow rate of 1 mL/min. A-100% for 3 min, changed to A-50% over 10 min and maintained for 10 min, changed A-30% over 3 min and returned to A-100% over 4 min).

ITLC-SG (instant thin layer chromatography-silica gel) was also carried out for the assessment of ^99m^Tc-iFAP radiochemical purity. Three mobile phases were used, the 2-butanone phase was employed for the identification of free ^99m^TcO_4_^−^ (R_f_ = 1), where ^99m^Tc-iFAP, ^99m^Tc-EDDA/tricine and ^99m^Tc-colloid remained in the origin (R_f_ = 0); 0.1 M sodium citrate (pH 5) identified free ^99m^TcO_4_ (R_f_ = 1) and ^99m^Tc-EDDA/tricine coligands (R_f_ = 1), where ^99m^Tc-iFAP and ^99m^Tc-colloid remained in the origin (R_f_ = 0); finally, the third phase was used for the determination of ^99m^Tc-colloid (R_f_ = 0) and consisted of ammonium acetate-methanol (1:1 *v*/*v*) ( ^99m^TcO_4_^−^, ^99m^Tc-iFAP and ^99m^Tc-EDDA run with the solvent front; R_f_ = 1).

#### 3.2.5. Biological Characterization

##### Cell Culture

The cells were cultured under sterile conditions in a laminar flow hood. The N30 cell line was cultured in a DMEM-F12 medium (ATCC^®^ 30-2006 ™). The media were supplemented with 10% fetal bovine serum (SBF, Biowest) and with 1% of a mixture of antibiotics (ampicillin, streptomycin) and antifungal agent (amphotericin) (Gibco). The cells were kept in an incubator at 37 °C, 5% CO_2_ and with a saturated humidity. To carry out each experiment, the cells were detached from the surface of the culture boxes with 0.05% trypsin, centrifuged at 1500 rpm for 4 min and seeded again.

Normal fibroblasts (human dermal fibroblasts) were originally obtained from circumcision surgeries of pediatric foreskin at the “Facultad de Ciencias Químicas, Universidad Autónoma de San Luis Potosí, Mexico” [33]. In brief, the epidermis was separated from the dermis using dispase (Sigma-Aldrich) for 8 h; the dermis was treated for 4 h with collagenase I (Worthington Biochemical) to obtain the fibroblasts. Cells were cultured with a DMEM-F12 medium supplemented with 10% BFS (Gibco) and 1% penicillin/streptomycin (Gibco), and maintained in an incubator at 37 °C, 83% humidity and 5% CO_2_. Fibroblasts used in the assays were passage 3 [33].

##### Western Blot

N30 and normal fibroblast cells were scraped off, transferred to a microfuge tube and lysed using ProteoJET^TM^ Mammalian Cell Lysis Reagent (Fermentas Life Sciences, Waltham, MA, USA). After centrifugation at 20,000× *g* for 15 min and protein quantification, polyacrylamide gel electrophoresis (SDS-PAGE) was performed using 30 µg of protein from each sample. The proteins were transferred to PVDF membranes (Merck Millipore) and blocked for 1 h at room temperature using PBS with 5% bovine serum albumin. The antibodies used for the Western blot were: FAP monoclonal antibody (Invitrogen F11-24; 1:1000) and GAPDH (GeneTex, 1: 5000). The primary antibodies were incubated overnight at 4 °C and subsequently washed. The species-specific HRP-conjugated secondary antibodies were incubated for 1 h at room temperature and washed twice. The membranes were incubated with the Super Signal West Femto substrate (Thermo Fisher Scientific, Waltham, MA, USA) and the signals were detected using the In Vivo Xtreme imaging system (Bruker, Billerica, MA, USA).

##### Cellular Uptake and Internalization

The ^99m^Tc-iFAP uptake and internalization by N30 (FAP +) and fibroblast (FAP −) cells were determined by using 4 × 10^5^ cells/500 µL of PBS in test tubes (n = 6). Both cells lines were incubated for 1 h at 37 °C with 10 µL of ^99m^Tc-iFAP (37 kBq, 0.4 nmol iFAP). After incubation, three washes with 500 µL of PBS were performed by centrifugation (700 g/5 min). By using a crystal scintillation (NaI(Tl)) well-type detector (MNL Inc., Littleton, CO, USA), the radioactivity in the cell pellet was evaluated. A standard volume with the initial activity was used to represent 100%, and the cell uptake activity was assessed with regard to this value. For the evaluation of internalization, the cell pellets were treated at 37 °C for 5 min with 500 µL of an acid glycine solution (0.1 M glycine, pH adjusted to 2.8 with HCl), and centrifuged (700 g/5 min). The cell pellet was incubated with 1 M NaOH for 5 min and centrifuged (700 g/5 min). The radioactivity of the supernatant was calculated as the percentage of internalization with regard to the initial activity of the standard volume as mentioned above.

As a qualitative evidence of iFAP recognition by FAP expressed in N30 cells, the iFAP ligand was incubated for 30 min at 37 °C with S-2-(4-isothiocyanatobenzyl)-1,4,7,10-tetraazacyclododecane tetraacetic acid (p-SCN-Bn-DOTA, Macrocyclics USA, Co., Plano, TX, USA) to be covalently attached on the surface of lutetium oxide nanoparticles (Lu_2_O_3_-iFAP colloidal solution) through DOTA groups. The nanoparticle size was determined by DLS and the DOTA conjugation was confirmed by FT-IR spectroscopy, as we previously reported [32]. N30 cells were then incubated with Lu_2_O_3_-iFAP or Lu_2_O_3_ (2 h at 37 °C), fixed in acetone and washed with PBS, followed by the addition of Hoechst (DNA stain, 250 μL,1μg/mL, and rinsed with PBS) to be observed in an epifluorescent microscopic field of 40×.

##### Human Serum Stability and Protein Binding

The ^99m^Tc-iFAP (37 MBq, 100 µL, 14 nmol) was incubated at 37 °C with 500 µL of human serum (IST-909c certified human serum sample, Sigma-Aldrich) and diluted in 0.5 mL of a 0.9% NaCl solution. Twenty microliters of the mixture were sampled at 30 min, 3 and 24 h for size-exclusion radio-HPLC analysis using a ProteinPak 300SW Waters column and 0.01M PBS as the mobile phase (1 mL/min). The percentage of recovery (quantification of the radioactivity recovered from the HPLC column) was determined by injecting 20 µL of the ^99m^Tc-iFAP sample into the HPLC system and 20 µL into a 50 mL volumetric flask, which was filled with 0.01 M PBS (flask 1). The column was eluted with 0.01 M PBS for 50 min (1 mL/min) and the effluent was collected in a 50 mL volumetric flask (flask 2). Aliquots of 1 mL from both flask 1 and flask 2 were measured on an HPGe detector (ORTEC). The percentage of recovery was calculated by dividing the counts in flask 2 by the counts in flask 1 then multiplied by 100.

##### Biodistribution and Imaging

The biodistribution studies in Balb-c male mice were carried out in agreement with Official Mexican Norm 062-ZOO-1999. Hep-G2 tumors were induced in mice (*n* = 15) by subcutaneous injection (right thigh; positive control) of 1 × 10^6^ cells suspended in 0.2 mL of phosphate-buffered saline. Killed Klebsiella pneumoniae bacteria (heated for 2 h at 1000 °C and verified for absence of growth after culturing), aseptically suspended in 0.2 mL of PBS (1 × 10^6^ bacteria), were subcutaneously administered into the left thigh of mice in order to induce inflammation (negative control). After the tumor was visible, ^99m^Tc-iFAP (50 µL, 18 MBq, 7 nmol of iFAP) was administered intravenously into the tail of each mouse. Three mice were selected for micro-SPECT/CT imaging, whereas the rest were sacrificed to be used in the biodistribution study. The times of evaluation were 15 min, 30 min, 2 h and 24 h. After the mice were sacrificed, the blood and main organs were removed, weighted and their radioactivity measured using a Na(Tl) detector, in order to calculate the percentage of the injected dose per gram of tissue (%ID/g) by using an initial standard volume (100% of the initial injected activity). Biokinetic models of the four main target tissues/organs (blood, tumor, liver and kidneys) were obtained by fitting ^99m^Tc-iFAP time-activity curves to functions with two or three exponential terms Equation (2):

(2) $$A\left(t\right)=Ae^{-at}+Be^{-bt}+Ce^{-ct}\;\;\;\;\;$$

Tumor imaging and tumor standard uptake value (SUV) calculations using Equation (3) were performed by obtaining micro-SPECT/CT images (ONCOVISION, Albira, Spain) at 30 min and 24 h from mice under 2% isoflurane anesthesia:

(3) $$SUV=\frac{decay\;corrected\;activity\;\left(MBq\right)\;in\;the\;tumor/tumor\;mass\;\left(g\right)}{total\;injected\;activity\;\left(MBq\right)/mice\;weight\;\left(g\right)}\;\;$$

## 4. Conclusions

The ^99m^Tc-iFAP, designed and synthesized for this research, shows suitable properties as a new FAP inhibitor radioligand based on the ^99m^Tc-HYNIC-D-alanine-boroPro structure for SPECT tumor microenvironment imaging. The findings of the analyses indicate high radiotracer stability in human serum (>95% at 24 h), specific recognition for FAP, high tumor uptake (7.05 ± 1.13% ID/g at 30 min) and fast kidney elimination. The results found in this study justify additional dosimetric and clinical studies in order to establish the sensitivity and specificity of ^99m^Tc-iFAP.

## Figures and Tables

**Table 1:** Affinity scores determined by molecular coupling (AutoDock), as well as the inhibition constants (*Ki*), and interaction distance between the boron residue and Serine 624 of the fibroblast activation protein (FAP) for each boroPro ligand.

Ligand: boroPro	Affinity (kcal/mol)	*Ki* (nM)	Distance Ser624-Boro (Å)
**N-acyl-Gly-boroPro** 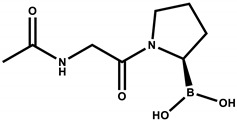	−4.80	296.46	3.398
**N-(pyridine-4-carbonyl)-D-Ala-boroPro)** 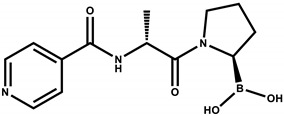	−6.60	14.07	3.657
**iFAP** 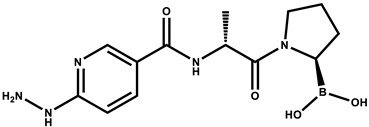	−8.53	0.536	3.274

**Table 2:** Biodistribution of ^99m^Tc-iFAP in mice with induced Hep-G2 tumors, indicated as a percentage of injected dose per gram of tissue (%ID/g).

Tissue	Time			
	15 min	30 min	2 h	24 h
Blood	2.07 ± 0.63	1.02 ± 0.32	0.33 ± 0.05	0.01 ± 0.01
Heart	1.75 ± 0.08	0.88 ± 0.23	0.19 ± 0.03	0.00
Lung	1.11 ± 0.51	0.65 ± 0.19	0.02 ± 0.01	0.00
Liver	2.78 ± 0.42	2.14 ± 0.51	1.05 ± 0.29	0.05 ± 0.03
Pancreas	0.64 ± 0.10	0.31 ± 0.12	0.12 ± 0.04	0.00
Spleen	1.07 ± 0.17	0.82 ± 0.04	0.15 ± 0.05	0.00
Kidneys	13.85 ± 2.27	11.18 ± 1.54	7.46 ± 1.02	1.89 ± 0.53
Intestine	1.42 ± 0.74	0.93 ± 0.14	0.22 ± 0.10	0.00
Stomach	0.71 ± 0.08	0.39 ± 0.12	0.25 ± 0.03	0.00
Muscle (left thigh)	0.39 ± 0.15	0.31 ± 0.11	0.18 ± 0.06	0.00
Bone	0.20 ± 0.09	0.14 ± 0.06	0.13 ± 0.02	0.00
Tumor	9.87 ± 1.94	7.05 ± 1.13	5.18 ± 0.82	2.37 ± 0.26
